# Translation and Validation of the Functionality Satisfaction and the Functionality Intent Scales in a French Sample

**DOI:** 10.3390/ejihpe16090137

**Published:** 2026-09-09

**Authors:** Eva Roy, Alain Guerrien, Claire Arnaud, Rachel F. Rodgers, Amélie Rousseau

**Affiliations:** 1 ULR 4072—PSITEC—Psychologie: Interactions Temps Émotions Cognition, University of Lille, F-59000 Lille, France; alain.guerrien@univ-lille.fr (A.G.); claire.arnaud@univ-lille.fr (C.A.); amelie.rousseau@univ-tlse2.fr (A.R.); 2 APPEAR, Department of Applied Psychology, Northeastern University, Boston, MA 02115, USA; r.rodgers@northeastern.edu; 3 Department of Psychiatric Emergency & Acute Care, Lapeyronie Hospital, CHRU Montpellier, 34090 Montpellier, France; 4 Unité de Recherche Clinique & Innovation, Hôpital du Cotentin, 50100 Cherbourg, France; 5 Centre d’Études et Recherches en Psychopathologie et Psychologie de la Santé (CERPPS), Université de Toulouse, UT2J, 31058 Toulouse, France

**Keywords:** body functionality, positive body image, scale adaptation, psychometric properties, measurement invariance, functionality appreciation

## Abstract

Body functionality is a promising target for the promotion of positive body image. However, research on body functionality in French-language contexts is currently limited due to the lack of validated assessment tools. This study aims to translate and evaluate the psychometric properties of the French versions of the Functionality Satisfaction Scale (FSS) and the Functionality Intent Scale (FIS). Exploratory factor analyses were conducted in a sample of 528 French adults (study 1), revealing a two-factor structure for both the FSS and FIS. Participants also completed validated measures of positive body image (functionality appreciation, body appreciation, and body esteem). A subsample (*n* = 150) completed the FSS and FIS again, three weeks later. Results supported convergent and divergent validity, good internal consistency, and satisfactory test–retest reliability. A second sample of 272 French adults was recruited for confirmatory factor analyses (study 2). Results confirmed the eight-item, two-factor solution for the FSS. However, support was found for a unidimensional structure of the FIS, which was ultimately retained as a four-item, one-factor scale. Measurement invariance analyses supported gender and age invariance up to the scalar level, but not at the strict level, for the FSS. The FIS also showed configural, metric, and scalar invariance across gender, whereas the scale was invariant across age only up to the metric level. This study provides support for the FSS as a useful tool for research focused on body functionality in French populations. Although the FIS demonstrated promising psychometric properties, it still requires further independent cross-validation to confirm its unidimensional structure.

## 1. Introduction

Historically, the field of body image has largely focused on negative dimensions (Bonfanti et al., 2025; Rodgers et al., 2023; Sharpe et al., 2018; Zhou et al., 2025). More recently, research attention has been paid to body image constructs grounded in positive psychology. As highlighted by numerous studies (Hallez et al., 2026; Iannattone et al., 2025; Linardon & Messer, 2026; Vankerckhoven et al., 2025), there is a crucial need for research focused on positive, adaptive, and healthy relationships with the body to complement the robust research on negative body image, in order to better understand body image in all its complexity. The present study therefore aimed to expand the available valid and reliable psychometric tools to assess positive body image among French-speaking groups.

Positive body image is a multidimensional construct, distinct from negative body image (Tylka & Wood-Barcalow, 2015b). It can be defined as appreciation and respect for the body, its functions, health, and characteristics (Williamson & Karazsia, 2018), allowing individuals to (1) recognize, accept, and love the uniqueness of their body, even when it does not align with beauty ideals, (2) pay attention to their body’s needs, and (3) protect themselves from negative body messages related to appearance (such as the portrayal of idealized appearances and promotion of diet culture, for instance; Guest et al., 2019; Tylka & Wood-Barcalow, 2015b; Wood-Barcalow et al., 2010). Positive body image is linked to adaptive behaviors (including self-care, intuitive eating, exercising without appearance-related motivations, and using skin protection) and is associated with both higher physical and psychological well-being (Brichacek et al., 2025; Halliwell, 2015; Linardon et al., 2022; Tylka & Wood-Barcalow, 2015b; Williamson & Karazsia, 2018). Positive body image may constitute an important protective factor against sociocultural appearance pressures and may decrease risk for health concerns, such as eating disorders, risky behaviours, or even depression (Carrard et al., 2019; Linardon et al., 2022). In this way, developing tools to assess and enhance positive body image represents an important opportunity.

One of the key components of positive body image is functionality (Alleva & Tylka, 2021; Halliwell, 2015). Body functionality refers to “everything that the body can do or is capable of doing” (Alleva & Tylka, 2021, p. 149). It is a multidimensional concept encompassing the diversity and the breadth of the body’s capabilities, including healing functions and internal mechanisms, senses, feelings, and sensibilities, physical and creative abilities, and self-care and relation with others (Alleva et al., 2015). Body functionality exists among all individuals and should not be limited to able-bodied individuals (Alleva & Tylka, 2021; Vinoski Thomas et al., 2019). The capacities of individuals with disabilities, or those limited by age or illness, will be different but not absent (Vinoski Thomas et al., 2019). Importantly, research on body functionality is grounded in a weight-neutral framework, which emphasizes body respect and appreciation regardless of body size or weight status (Alleva & Tylka, 2021). Within this perspective, functionality represents what the body can do, not how it looks or how much it weighs. The very broad applicability of this dimension of positive body image, and its variability, make it an interesting focus of research and a potential target for interventions tailored to populations at higher risk of developing negative body image and eating disorders (Vinoski Thomas et al., 2019; Ramesh et al., 2026).

Given the potential protective role of functionality and its connection to adaptive behaviours, research efforts have focused on developing interventions to target this dimension and tools capable of assessing it (see Alleva et al., 2014, 2018a, 2018b, 2020, 2015; Glashouwer et al., 2025; Granfield et al., 2023; Guest et al., 2024; Smith et al., 2023; Weiland et al., 2026; Weaver & Mulgrew, 2021). In particular, functionality appreciation as assessed through the Functionality Appreciation Scale (Alleva et al., 2017) has emerged as one of the most reliable and comprehensive measures of body functionality (Marmara & Zarate, 2026). However, functionality appreciation, defined as appreciation and respect for body functionality, represents only one facet of the broader concept of body functionality. Beyond functionality appreciation, other dimensions, such as functionality satisfaction and intention, are also central. Whereas functionality appreciation reflects a grateful, respectful, and accepting orientation toward the body and what it is capable of doing (Alleva et al., 2017), functionality satisfaction is conceptually narrower and reflects an evaluative judgment about how pleased an individual is with their specific body functions or overall body functionality. Thus, individuals may appreciate and respect their body despite being dissatisfied with some of its current functions (e.g., due to illness, injury, or ageing), highlighting that these constructs are conceptually related but distinct. Functionality intent, here, captures a person’s motivational intention to engage in cognitive and behavioural practices that direct attention toward, and deepen the experience of, body functionality. Importantly, due to the relative recency of this research domain, instruments to assess these dimensions are relatively new, and those that exist have largely been developed and validated among English-speaking groups. In particular, there is a lack of validated measurement tools in French, and especially a need for measures assessing state-like constructs. Thus, the validation of recent tools in the French language would constitute an important step towards incremental research on body functionality among French-speaking groups.

The aim of this current research was therefore to translate and validate two tools developed by Mulgrew and Courtney (2022): the Functionality Satisfaction Scale (FSS) and the Functionality Intent Scale (FIS). The eight-item Functionality Satisfaction Scale was developed to assess multiple dimensions of body functionality, including satisfaction, appreciation, and the capacity to use one’s body to significantly and profoundly interact with the environment and individuals. Importantly, a key strength of the FSS is that it assesses functionality satisfaction as a state-like construct, making it suitable for capturing short-term changes following interventions or daily experiences. The FSS was particularly useful to assess state changes in functionality satisfaction after experimental manipulations, such as exposure to functionality content through Instagram posts (Mulgrew & Courtney, 2022), body-positive content (Hepburn & Mulgrew, 2023), idealized images (Mulgrew & Tiggemann, 2018), or completion of writing tasks (Mulgrew et al., 2017; Mulgrew & Boyer, 2024) or multi-session program (Mulgrew et al., 2019; Thøgersen-Ntoumani et al., 2022). The initial exploratory psychometric work revealed a unidimensional structure. The scale showed good internal consistency at both timepoints assessed (α = 0.93, α = 0.96). However, as highlighted by Longhurst et al. (2025), this tool has not yet been submitted to deeper psychometric evaluations.

The Functionality Intent Scale comprises four items evaluating the intention of individuals to engage in meaningful functional activities. It also showed good internal consistency across timepoints (α = 0.81, α = 0.90). The development of psychometrically sound French-language versions of both of these scales would support research examining these dimensions in French-speaking populations and the assessment of interventions aiming to promote body functionality. The validation of these scales in the French language will represent a significant contribution to supporting the examination of body functionality within the French cultural context and ensuing interventions targeting body functionality. Furthermore, these scales will offer healthcare professionals empirically validated instruments for assessing positive body image and associated adaptive behaviours. To achieve these aims, two empirical studies were conducted. The first study investigated the factorial structure of the two scales and assessed their validity and reliability. The analyses were fully exploratory in the French sample; the study examined the factorial structure of the scales without imposing the original factor structures. The second study employed confirmatory analyses to validate the factor structures identified in study 1, thereby supporting the robustness and applicability of these instruments for both research and clinical practice.

## 2. Study 1

### 2.1. Study 1 Method

#### 2.1.1. Participants

Five hundred and twenty-nine participants took part in the study. One participant’s data were excluded because they did not meet the age inclusion criterion (minimum 18 years). Consequently, analyses were conducted on 528 participants (75.8% women, 20.8% men, and 3.4% persons of diverse genders including non-binary, genderfluid, or agender individuals). The mean age was 28.4 years (±12). The data showed an average Body Mass Index (BMI) of 23.8 (±4.93). Regarding body weight satisfaction, 13.5% wanted to gain weight (7.3% women and 6.1% men), 72.3% wanted to lose weight (57.8% women and 12.0% men), and 14.1% were satisfied with their current weight (10.5% women and 2.9% men). Most of the sample (65.1%) engaged in physical activity at least once a week, and 30.6% engaged in a body-based artistic activity at least once a week. The main stated reasons for taking part in physical activity were physical health, mental health, and feeling good about their body, whereas the main motivation for taking part in artistic activities was mental health. Physical-appearance-related motivations were more important for physical activity (33.5%) than for artistic activity (2.1%).

Participants were recruited through social media (Facebook, LinkedIn, Instagram posts) and through posters on the University of Lille campus. To minimize response bias, participants were initially informed that the study examined positive body image and well-being (rather than the specific objective of validating body functionality questionnaires). After data collection, all participants received a comprehensive debriefing explaining the study’s detailed objectives and providing contact information for mental health resources. This procedure was approved by the University Ethics Committee (reference code: 2023-755-S123). All participants provided informed consent prior to participation. Inclusion criteria were proficiency in the French language and an age of at least 18 years.

Sample size was determined based on commonly accepted recommendations for psychometric validation studies (Costello & Osborne, 2005; Swami & Barron, 2019; Worthington & Whittaker, 2006). With 528 participants and an estimated 12 items total across the two scales (FSS: eight items, FIS: four items), the sample provided 44 participants per item, exceeding the commonly recommended ratio of 5–10 participants per item.

#### 2.1.2. Materials

##### Scales Undergoing French-Language Validation

Functionality Satisfaction

The Functionality Satisfaction Scale (FSS; Mulgrew & Courtney, 2022) is a state self-report questionnaire comprising eight items designed to assess individuals’ evaluation of their body functionality across multiple dimensions encompassing satisfaction, appreciation, and the ability to use one’s body to connect with the world and others in a coherent manner. The scale includes items such as “Right now, I feel respect for the many unique and varied tasks my body performs” and “Currently, I respect that my body has the ability to help me to rest, sleep, and restore.” Participants are asked to reflect on how they feel at the present moment and respond by indicating their mood on a visual analog scale ranging from “not at all” (0) to “completely” (100). An average of the scores from the eight items is calculated. Higher scores indicate a greater level of functional satisfaction. The original version revealed a unidimensional structure and good internal consistency (α = 0.93, α = 0.96).

Functionality Intent

The Functionality Intent Scale (FIS; Mulgrew & Courtney, 2022) is a self-report questionnaire that includes four items assessing intent to experience body functionality. The purpose of this scale is to assess participants’ intentions to engage in activities that promote the experience of body functionality, with items such as “I intend to be attentive to what my body needs” and “I intend to engage in self-care and other meaningful activities that help me to look after my body and mind.” Participants are asked to reflect on how they feel at the moment and respond by indicating their mood on a visual analog scale ranging from “not at all” (0) to “completely” (100). An average of the scores from the four items is calculated. Higher scores indicate greater functionality-related intentions. Good internal consistency was found in the original version (α = 0.81, α = 0.90).

##### Scales Used for the Investigation of the Psychometric Properties

Functionality Appreciation Scale

The Functionality Appreciation Scale (FAS; Alleva et al., 2017; French version: Arnaud et al., 2025) measures overall appreciation of the body’s ability to function within its possibilities. It is a self-report questionnaire consisting of seven items, such as “I am grateful for the health of my body, even if it isn’t always as healthy as I would like it to be” and “I feel that my body does so much for me.” The aim is to measure whether participants appreciate, respect, and value their bodies for what they are capable of doing. Participants respond on a five-point Likert scale ranging from 1 (strongly disagree) to 5 (strongly agree). An average score is calculated across the items. Higher scores show a greater level of appreciation for body functionality. In this study, McDonald’s omega was 0.85.

Body Esteem Scale

The Body Esteem Scale (BES; Mendelson et al., 2001; French version: Rousseau et al., 2015) is a self-report questionnaire that assesses attitudes and feelings regarding the body and the appearance through 23 items divided into three subscales of body esteem. Ten items constitute the “Appearance” subscale and measure the overall feelings regarding physical appearance. Eight items evaluate how satisfied or dissatisfied participants feel with their weight, representing the “Weight” subscale. Finally, the “Attribution” subscale consists of five items that assess beliefs about how others perceive their body appearance. Participants answer on a five-point Likert scale (never, rarely, sometimes, often, always). Higher scores indicate a greater level of body esteem and a more positive evaluation of appearance. McDonald’s omega was 0.95.

Body Appreciation Scale-2

The Body Appreciation Scale-2 (BAS-2; Tylka & Wood-Barcalow, 2015a; French version: Kertechian & Swami, 2017) is a self-report questionnaire designed to evaluate body image without emphasizing issues like thinness or muscularity. Unlike other body image scales, the BAS-2 is not gender-specific and does not contain items that are linked to sex or gender norms. It measures body appreciation through 10 items, such as “I appreciate the different and unique characteristics of my body.” Participants rate each item on a five-point Likert scale (never, rarely, sometimes, often, always). Higher scores reflect a greater level of positive body image. McDonald’s omega was 0.93 in the current study.

Socio-demographic questionnaire

Participants were asked to indicate their age, gender (men, women, or other with write-in option), height, and current weight.

#### 2.1.3. Procedure

##### Translation into French

We requested and received permission from the authors to translate and validate the original versions of the Functionality Satisfaction Scale and the Functionality Intent Scale into French. The French versions were produced through forward translation and expert consensus review. The process involved five steps. First, two bilingual translators (fluent in English and French, both with expertise in body image) independently translated the items into French. Second, the two translated versions were compared and reconciled into a preliminary synthesis. Third, a bilingual researcher in psychology reviewed the synthesized version. Fourth, two additional psychology researchers (both proficient in English, one specialist in the field of body image) reviewed and discussed the items. Fifth, the research team finalized the French versions through consensus-based modifications. These modifications ensured semantic (word-for-word accuracy) and conceptual equivalence (maintaining the intended meaning and psychological construct). For example, the sentence “to be attentive” was translated as “d’être attentif/attentive” rather than “faire attention à”, to preserve the intended linguistic and phenomenological nuance.

##### Scale Testing

This study received approval from the University’s Board of Ethics (reference code: 2023-755-S123, VFEF, validated on 21 February 2024). The study took place online, using the LimeSurvey platform, and recruitment occurred from 1 March 2024, to 2 July 2024. The link was shared on social media, with an advertisement mentioning that the study focused on positive body image and well-being. The inclusion criteria were specified (over 18 years old and fluent in French). Potential participants could click on the link to access the study page and provide informed consent before accessing the survey.

To gather test–retest data, each participant was required to create a unique code that was then used to link their responses across surveys. Participants were invited to complete the questionnaires in the following order, namely the Functionality Satisfaction Scale, the Functionality Intent Scale, the FAS, the BAS-2, the BES, the physical and artistic activities questionnaire, and the socio-demographic questionnaire.

At the end of the assessment, the participants were thanked and invited to provide their email address via a separate link so they could be contacted three weeks later for the retest phase of the study. One hundred and fifty volunteers participated three weeks later and completed the Functionality Satisfaction Scale and the Functionality Intent Scale. After completing the questionnaire, participants were debriefed and resources for healthcare professionals were provided.

#### 2.1.4. Statistical Analyses

The exploratory analyses were conducted using Jamovi Version 2.5.6 (The Jamovi Project, 2024). Initially, the skewness and kurtosis indices were checked to evaluate the normality of the data. All items of each scale (Functionality Satisfaction and Functionality Intent) showed skewness and kurtosis values within the range of −1 to 1, indicating that the distributions were reasonably normally distributed (Gaskin & Happell, 2014; Mishra et al., 2019; Tabachnick & Fidell, 2007). The scores of the other questionnaires (FAS, BAS-2, and BES) also met normality requirements. To determine the suitability of the data for factor analyses, inter-item correlations and item-total correlations (which should at least be >0.15, and ideally >0.40–0.50, Clark & Watson, 1995), the Kaiser–Meyer–Olkin (KMO) measure of sampling adequacy (which should be ≥0.60 and ideally ≥0.80; Kaiser, 1974), and Bartlett’s test of sphericity (which should be significant; Bartlett, 1950) were calculated. Exploratory factor analyses (EFAs) were then conducted to explore the factor structure of our scales. As is common practice in body image research (Swami & Barron, 2019) and per the initial evaluation of the Functionality Satisfaction measure (Mulgrew & Courtney, 2022), a principal-axis factoring was performed, using a direct oblimin rotation. The number of factors extracted was based on parallel analysis (Horn, 1965), as it has been demonstrated to be one of the most precise and statistically robust methods for determining the number of factors to retain (Hayton et al., 2004; Henson & Roberts, 2006; Swami & Barron, 2019; Swami et al., 2021a; Zwick & Velicer, 1986). A cut-off of 0.40 was used for item loading (Howard, 2016; Reio & Shuck, 2015).

Internal consistency was assessed using McDonald’s omega (Dunn et al., 2014). Pearson’s correlation coefficient was used to assess the stability of the scores from Time 1 to Time 2 (three weeks later). Also, as recommended by Swami and Barron (2019), an analysis of the intraclass correlation coefficient (ICC) was performed to evaluate test–retest reliability, based on a mean-rating (*k* = 2), absolute-agreement, two-way model. ICC was interpreted using a 95% confidence interval (Koo & Li, 2016). Values under 0.50 indicate poor reliability, values between 0.50 and 0.75 suggest moderate reliability, values between 0.75 and 0.90 indicate good reliability, and values over 0.90 show excellent reliability (Koo & Li, 2016). Finally, a Kolmogorov–Smirnov test was performed to measure the normality of our sample, allowing us to conduct paired-sample *t*-tests to measure mean-level stability.

Convergent validity was tested by examining the relationships between the scales of interest and the other measures of positive body image (FAS, BAS-2, and BES). Consistent with previous work, divergent validity was assessed by examining the association with BMI (Alleva & Tylka, 2021; Alleva et al., 2017; Arnaud et al., 2025). Indeed, BMI is not conceptualized as an indicator of body functionality, which is grounded in a weight-neutral framework. In addition, as a supplementary analysis, correlations with age were also tested to gain preliminary insight into potential age-related patterns in body functionality perceptions, thereby contributing to a broader understanding of how body functionality operates across the lifespan. There were no missing data, except for 4 data points for BMI. Given the number of correlations conducted to assess convergent and divergent validity, Benjamini–Hochberg correction for false discovery rate was applied within each family of tests (correlations with positive body image measures, BMI, and age, respectively).

### 2.2. Study 1 Results

#### 2.2.1. Functionality Satisfaction

Item analysis and exploratory factor analysis. Adequate inter-item correlations (ranging from *r* = 0.39, CI 95% [0.32; 0.47], *p* < 0.001 to *r* = 0.80, CI 95% [0.76; 0.83], *p* < 0.001) and high item-total correlation coefficients were found at time 1, ranging from *r* = 0.66, CI 95% [0.61; 0.71], (*p* < 0.001) to *r* = 0.86, CI 95% [0.84; 0.88] (*p* < 0.001) (see Supplementary Materials, Table S1). The Kaiser–Meyer–Olkin measure of sampling adequacy (KMO = 0.92) and the significance of Bartlett’s test of sphericity, *χ*^2^(28) = 2980, *p* < 0.001, confirmed that the data were suitable for EFA. Parallel analysis was used in order to determine the number of factors retained (Horn, 1965). The results revealed a two-factor model. The first factor, called “Satisfaction with body capacities” (λ1 = 2.94), accounted for 36.8% of the common variance. Four items loaded on this factor significantly (ranging from 0.47 to 0.95, see Table 1). The second factor, called “Gratitude for body functions and worldly connection” (λ2 = 2.39), accounted for 29.9% of the common variance. Five coefficients were greater than 0.40 (ranging from 0.42 to 0.86). However, given that item 7 showed a higher loading on the first factor (0.47), it was allocated to the first factor, which aligned with the theme of satisfaction regarding the body’s capacities, despite also having a moderate loading on the second factor (0.42). In this way, four items constituted the second factor.

Reliability of the Functionality Satisfaction Scale. Participants who completed the retest and those who did not were compared. They did not significantly differ on age, gender, BMI, or pre-test measures of the outcomes. Internal consistency was strong. McDonald’s omega values were ω = 0.92 at time 1, and ω = 0.95 at time 2 for global scores (Factor 1: ω = 0.92 at time 1, ω = 0.95 at time 2; Factor 2: ω = 0.83 at time 1, and ω = 0.88 at time 2). Test–retest reliability over a 3-week period was examined in a subsample (*n* = 150). Global scores showed strong positive correlations with *r* = 0.80 (*p* < 0.001; *n* = 150), CI 95% [0.74; 0.85]. Each factor also demonstrated good stability (Factor 1: *r* = 0.81, CI 95% [0.75; 0.86], *p* < 0.001; Factor 2: *r* = 0.74, CI 95% [0.66; 0.80], *p* < 0.001). Intraclass correlation coefficient (ICC; *k* = 2, absolute-agreement, two-way model) confirmed acceptable reliability for global scores (*ICC* = 0.80, CI 95% [0.74; 0.85]) and for each factor (Factor 1: *ICC* = 0.81, CI 95% [0.75; 0.86]; Factor 2: *ICC* = 0.74, CI 95% [0.67; 0.80]). As normality was verified (all *K-S* values < 0.10, *p* > 0.13), paired-sample *t*-tests examined mean-level stability. Results were non-significant for global scores, *t*(149) = −0.57, *p* = 0.57, and *d* = −0.047, and for each factor, with *t*(149) = −0.04, *p* = 0.97, and *d* = −0.003 for Factor 1 and *t*(149) = −0.92, *p* = 0.36, and *d* = 0.075 for Factor 2, indicating good mean-level stability.

Convergent validity. Higher Functionality Satisfaction scores were strongly associated with higher levels of functionality appreciation (FAS; *r* = 0.72, CI 95% [0.67; 0.76], *p* < 0.001, BH-adjusted *p* < 0.001), body appreciation (BAS-2; *r* = 0.68, CI 95% [0.63; 0.72], *p* < 0.001, BH-adjusted *p* < 0.001), and body esteem (BES; *r* = 0.59, CI 95% [0.53; 0.64], *p* < 0.001, BH-adjusted *p* < 0.001).

Incremental validity. Given that functionality satisfaction is conceptually related to, yet theoretically distinct from, functionality appreciation, we examined whether Functionality Satisfaction accounted for unique variance in body appreciation and body esteem, above and beyond functionality appreciation. Multicollinearity diagnostics indicated no concerns (VIFs = 2.06). Incremental validity was examined using hierarchical regression analyses predicting body appreciation (BAS-2 scores) and body esteem (BES scores). In the first step, functionality appreciation (FAS) accounted for a substantial proportion of variance in BAS-2 (*R*^2^ = 0.48, *F*(1, 526) = 479.00, *p* < 0.001) and BES (*R*^2^ = 0.30, *F*(1, 526) = 230.00, *p* < 0.001) scores. In the second step, Functionality Satisfaction (FSS) was added to the model, resulting in a significant increase in explained variance (BAS-2: Δ*R*^2^ = 0.07, Δ*F*(1, 525) = 80.40, *p* < 0.001; BES: Δ*R*^2^ = 0.08, Δ*F*(1, 525) = 63.70, *p* < 0.001). In the final model, both FAS (BAS-2: *β* = 0.42, CI 95% [0.34; 0.50], *p* < 0.001; BES: *β* = 0.27, CI 95% [0.17; 0.37], *p* < 0.001) and FSS (BAS-2: *β* = 0.38, CI 95% [0.30; 0.46], *p* < 0.001; BES: *β* = 0.39, CI 95% [0.30; 0.49], *p* < 0.001) were significant predictors, indicating that FSS explained unique variance beyond FAS.

Divergent validity. Higher Functionality Satisfaction scores were weakly associated with lower levels of BMI (*r* = −0.23, CI 95% [−0.31; −0.15], *p* < 0.001, BH-adjusted *p* < 0.001). A weak correlation was found with age (*r* = 0.09, CI 95% [0.004; 0.17], *p* < 0.05, BH-adjusted *p* < 0.047). The results are available in Supplementary Materials (see Table S2).

#### 2.2.2. Functionality Intent

Item analysis and exploratory factor analysis. Adequate inter-item correlations (ranging from *r* = 0.32, *p* < 0.001 to *r* = 0.64, *p* < 0.001) and high item-total correlation coefficients were found at time 1, ranging from *r* = 0.73 (*p* < 0.001) to *r* = 0.86 (*p* < 0.001), revealing that the data met assumptions for EFA (see Supplementary Materials, Table S3). The Kaiser–Meyer–Olkin measure of sampling adequacy (KMO = 0.77) and the significance of Bartlett’s test of sphericity, *χ*^2^(6) = 729, *p* < 0.001, confirmed this. Again, parallel analysis revealed a two-factor structure: Factor 1, “Functionality Awareness” (λ1 = 1.27), and Factor 2, “Functionality Investment” (λ2 = 1.14), with the two factors accounting for 31.9% and 28.4% of the common variance, respectively. The four items loaded on these factors significantly (ranging from 0.53 to 0.80, see Table 2).

Reliability of the Functionality Intent Scale. Internal consistency analyses showed high McDonald’s omega values at times 1 and 2, with ω = 0.82 at time 1, and ω = 0.89 at time 2 for global scores (Factor 1: ω = 0.71 at time 1, and ω = 0.83 at time 2; Factor 2: ω = 0.72 at time 1, and ω = 0.77 at time 2). The test–retest reliability of the Functionality Intent Scale was assessed over a three-week period. Time 1 and time 2 scores showed strong positive correlations with *r* = 0.77 (*p* < 0.001; *n* = 150) for global scores, with a confidence interval between *r* = 0.69 (*p* < 0.001; *n* = 150) and *r* = 0.82 (*p* < 0.001; *n* = 150). Strong positive correlations between time 1 and time 2 were found for each factor (Factor 1: *r* = 0.76, CI 95% [0.68; 0.82], *p* < 0.001; Factor 2: *r* = 0.72, CI 95% [0.64; 0.79], *p* < 0.001). An analysis of the intraclass correlation coefficient (ICC) was performed based on a mean-rating (*k* = 2), absolute-agreement, two-way model. The results indicate good reliability (*ICC* = 0.77), ranging from 0.69 to 0.82. Acceptable reliability was found for each factor (Factor 1: *ICC* = 0.76, CI 95% [0.68; 0.82]; Factor 2: *ICC* = 0.72, CI 95% [0.64; 0.79]). As the normality requirements were met (global scores: *K-S* = 0.10, *p* = 0.09; Factor 1: *K-S* = 0.10, *p* = 0.10; Factor 2: *K-S* = 0.10, *p* = 0.13), a paired-sample *t*-test was performed and revealed non-significant results, with *t*(149) = 0.16, *p* = 0.87, and *d* = 0.013 for global scores, *t*(149) = −0.42, *p* = 0.68, and *d* = −0.034 for Factor 1, and *t*(149) = 0.69, *p* = 0.49, and *d* = −0.056 for Factor 2, supporting mean-level stability.

Convergent validity. Strong associations between higher Functionality Intent scores and higher levels of functionality satisfaction (FSS; *r* = 0.56, CI 95% [0.50; 0.62], *p* < 0.001, BH-adjusted *p* < 0.001), functionality appreciation (FAS; *r* = 0.52, CI 95% [0.46; 0.58], *p* < 0.001, BH-adjusted *p* < 0.001), body appreciation (BAS-2; *r* = 0.55, CI 95% [0.48; 0.60], *p* < 0.001, BH-adjusted *p* < 0.001), and body esteem (BES; *r* = 0.42, CI 95% [0.34; 0.49], *p* < 0.001, BH-adjusted *p* < 0.001) were found.

Incremental validity. Multicollinearity diagnostics indicated no concerns (VIFs = 1.38). Incremental validity was examined using hierarchical regression analyses predicting body appreciation (BAS-2 scores) and body esteem (BES scores). As previously mentioned, in the first step, FAS accounted for a substantial proportion of variance in BAS-2 (*R*^2^ = 0.48, *F*(1, 526) = 479.00, *p* < 0.001) and BES (*R*^2^ = 0.30, *F*(1, 526) = 230.00, *p* < 0.001) scores. In the second step, Functionality Intent (FIS) was added to the model, resulting in a significant increase in explained variance (BAS-2: Δ*R*^2^ = 0.05, Δ*F*(1, 525) = 51.40, *p* < 0.001; BES: Δ*R*^2^ = 0.02, Δ*F*(1, 525) = 17.80, *p* < 0.001). In the final model, both FAS (BAS-2: *β* = 0.56, CI 95% [0.49; 0.63], *p* < 0.001; BES: *β* = 0.46, CI 95% [0.38; 0.54], *p* < 0.001) and FIS (BAS-2: *β* = 0.25, CI 95% [0.18; 0.32], *p* < 0.001; BES: *β* = 0.18, CI 95% [0.10; 0.26], *p* < 0.001) were significant predictors, indicating that FIS explained unique variance beyond FAS.

Divergent validity. No significant correlations with BMI emerged. However, a significant weak correlation was found with age (*r* = 0.12, CI 95% [0.03; 0.20], *p* < 0.01, BH-adjusted *p* < 0.007; see Supplementary Materials, Table S2): the older the individuals, the higher their scores on functionality intent, indicating a greater intention to engage in body functionality.

### 2.3. Study 1 Discussion

This first study evaluated the psychometric properties of French translations of the Functionality Satisfaction Scale (FSS) and the Functionality Intent Scale (FIS), including their factor structures, reliability, and validity in a sample of French adults.

Findings from the EFA revealed that the eight items of the French FSS loaded significantly on two different factors, labelled “Satisfaction with body capacities” (four items) and “Gratitude for body functions and worldly connection” (four items). Despite being designed to capture the multiple facets of body functionality, the original scale development work had identified a one-factor solution (Mulgrew & Courtney, 2022), which is divergent from our findings. This discrepancy may suggest cultural differences in how body functionality satisfaction is conceptualized and experienced. However, confirmatory analyses are required to corroborate this two-factor solution. Overall, the scale showed good internal reliability, consistent with prior work (Mulgrew & Courtney, 2022; α = 0.93–0.96) and satisfactory test–retest reliability.

A two-factor model was identified for the FIS (four items), although each factor contains only two items, which may not be psychometrically adequate (Tabachnick & Fidell, 2013). Results provided preliminary evidence of good reliability and satisfactory internal consistency, consistent with previous findings (Mulgrew & Courtney, 2022), where good internal consistency at pre-test (α = 0.81) and post-test (α = 0.90) were described.

Both scales demonstrated strong convergent validity, as evidenced by significant correlations with positive body image measures (functionality appreciation, body appreciation, and body esteem). Hierarchical analyses supported the incremental validity of our scales relative to the FAS in predicting body appreciation and body esteem scores. Additionally, high intercorrelation among the scales of interest provided further evidence for validity (Swami et al., 2021a). Regarding divergent validity, no significant correlation with BMI was found for the FIS and weak inverse correlations emerged for the FSS. Although these correlations are weak and do not undermine the divergent validity, they warrant discussion. These relationships could reflect internalization of negative-weight stereotypes, wherein individuals with higher BMI may perceive reduced body functionality despite the holistic, weight-neutral, and inclusive framework on which this construct is grounded (Alleva & Tylka, 2021; Arnaud et al., 2025; Linardon et al., 2023). Future research should further explore the interplay among BMI, the internalization of weight-related stereotypes, and body functionality. Moreover, this finding highlights the need for interventions that explicitly address and decouple functionality from appearance-based judgments, reinforcing that functional appreciation is accessible and meaningful regardless of body size. Promoting a holistic and inclusive approach to body functionality may therefore be critical for fostering positive body image across diverse body types. In sum, study 1 provided promising support for the scales of interest, warranting their further examination using CFA in a different sample.

## 3. Study 2

### 3.1. Study 2 Method

#### 3.1.1. Participants

A sample of 272 adults was recruited to participate in this study (75.4% women, 21.3% men and 3.3% persons of diverse genders). The mean age was 31.2 years (±13.6) and mean BMI was 23.8 (±6.48). Inclusion criteria included proficiency in the French language and a minimum age of 18 years. Again, recruitment was conducted via social media. The differences between participants’ actual body weight and their ideal body weight revealed that 17.3% wanted to gain weight (7.7% women and 9.6% men), 65.9% wanted to lose weight (54.8% women and 8.5% men), and 16.9% were satisfied with their current weight, reporting the same actual and ideal body weight (12.9% women and 3.3% men).

This sample of 272 participants was considered adequate based on common recommendations suggesting samples of at least 100 or 200 participants and 5–20 observations per estimated parameter (Swami & Barron, 2019).

#### 3.1.2. Materials

The measures were identical to those employed in study 1.

#### 3.1.3. Procedure

This study received approval from the University’s Board of Ethics (reference code: 2023-755-S123, VFEF, validated on 21 February 2024). Except for those pertaining to the test–retest analysis, the procedures were identical to those in study 1. Recruitment occurred from 13 November 2024, to 7 April 2025.

#### 3.1.4. Data Analysis

The confirmatory analyses were conducted using Jamovi Version 2.5.6 (The Jamovi Project, 2024), with the SEMLj-SEM package based on lavaan for Confirmatory Factor Analysis (CFA; Rosseel, 2012). All items of each scale showed skewness and kurtosis values within the range of −1 to 1, confirming their normality (Mishra et al., 2019; Tabachnick & Fidell, 2007). Mardia’s coefficients were used to assess multivariate normality (Mardia, 1970). Significant results (*p* < 0.001 for both skewness and kurtosis) revealed that our data were multivariate non-normal. Considering our non-normal data and our sample size (Nalbantoğlu-Yılmaz, 2019), CFAs using the robust maximum likelihood method were conducted on the models identified in study 1. We also used the Satorra–Bentler scaled χ^2^ correction (Satorra & Bentler, 2001), as recommended by Swami and Barron (2019). The acceptability of model fit was examined using various fit indices: the χ^2^, which should be non-significant; the χ^2^/df ratio, which should be <3.0; the Comparative Fit Index (CFI) and the Tucker–Lewis Index (TLI), both of which should be at least 0.90 for acceptable fit and 0.95 for good fit; the root mean square error of approximation (RMSEA), with <0.08 indicating an acceptable fit and <0.05 indicating a good fit; and the standardized root mean square residual (SRMR), which should be less than 0.08 for a good fit (Hu & Bentler, 1999; Sathyanarayana & Mohanasundaram, 2024). Additionally, we used the chi-squared difference test to compare the models identified in study 1 with a one-factor structure for each scale (Brown, 2015). The item loading cut-off was set at 0.45 (Tabachnick & Fidell, 2007). There were no missing data.

The models were tested for measurement invariance across gender and age to ensure that the FSS and the FIS assessed the same construct across demographic groups. Multi-group CFAs were conducted at the configural, metric, scalar and strict levels, following the recommendations of Putnick and Bornstein (2016), using Jamovi Version 2.5.6 (The Jamovi Project, 2024) with the Rj Editor package (Love, 2018). The first step assessed configural invariance, without applying constraints to the model. Configural invariance was considered based on the model fit indices described previously. In a second step, factor loadings were constrained to be the same across groups in order to test metric invariance. Then, item intercepts were additionally constrained to be equal across groups to evaluate scalar invariance. In a final step, the residuals were constrained to be equal across groups to test strict invariance. Each model was compared to the previous one (metric compared to configural, scalar compared to metric, and strict compared to scalar) using multiple fit statistics differences in the Satorra–Bentler scaled chi-square (which should be non-significant), RMSEA, SRMR, CFI, and TLI. However, greater consideration was given to alternative fit indices (RMSEA, SRMR, CFI, and TLI) because the chi-square is overly sensitive to small samples (Chen, 2007; Putnick & Bornstein, 2016). We used criteria suggested by Chen (2007), whereby a decrease in robust CFI of ≥ 0.010 (ΔCFI ≤ −0.010), together with a change in robust ΔRMSEA ≥ 0.015 and robust ΔSRMR ≥ 0.030 (for metric invariance) or robust ΔSRMR ≥ 0.015 (for scalar and strict invariance) indicate non-invariance between groups.

### 3.2. Study 2 Results

#### 3.2.1. Functionality Satisfaction

**Figure 1:**
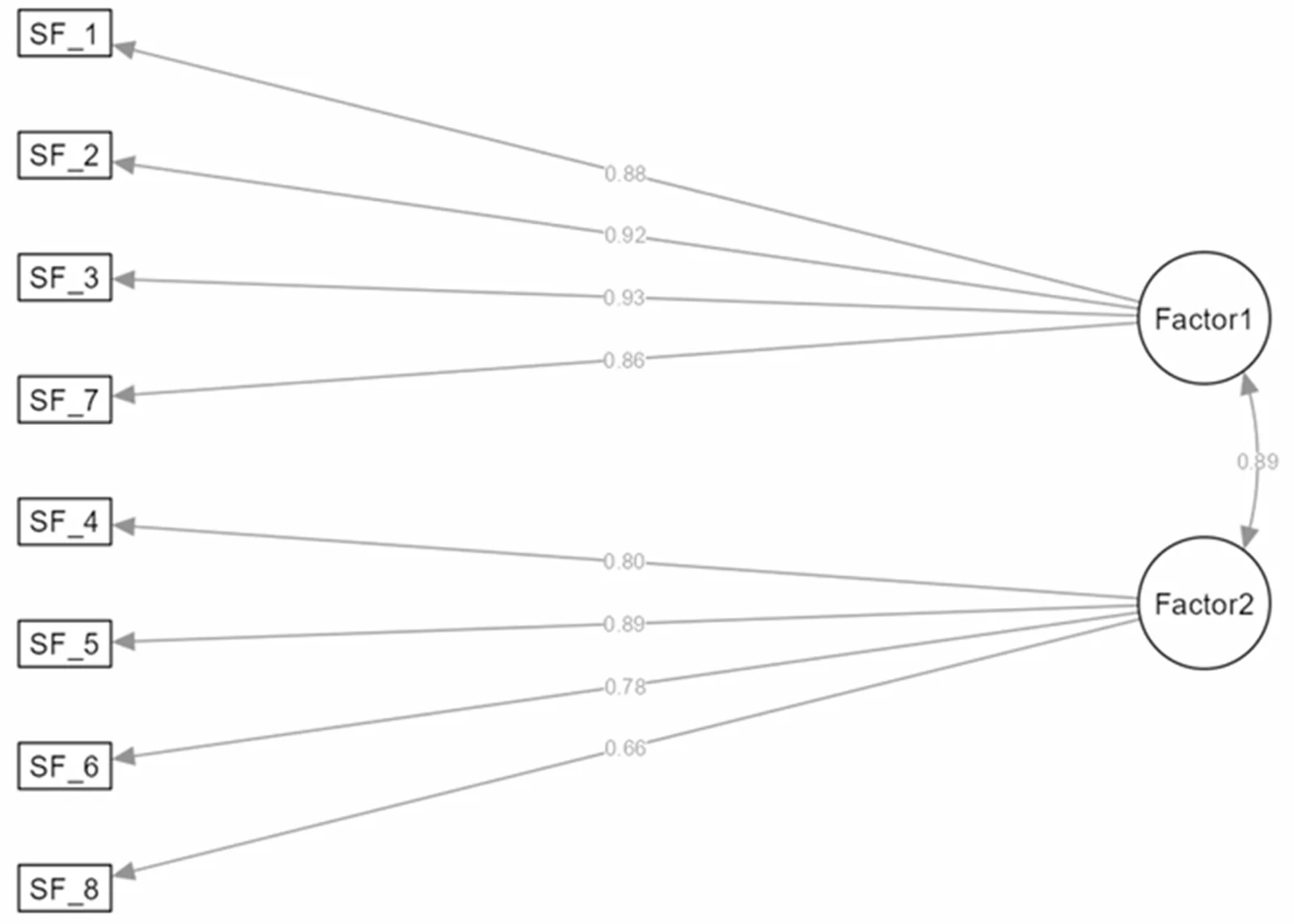
Path diagram and estimates for the two-factor structure of Functionality Satisfaction Scale scores in sample 2; Factor 1: “Satisfaction with body capacities”; Factor 2: “Gratitude for body functions and worldly connection”.

Confirmatory factor analysis. The SBχ^2^ was significant (SBχ^2^(1) = 136, *p* < 0.001), indicating a poor model fit. However, this index is sensitive to sample size (Sathyanarayana & Mohanasundaram, 2024). Taking the other fit indices into consideration, our model demonstrated an adequate fit to the data: SBχ^2^/df = 1.968; CFI = 0.987; TLI = 0.981; SRMR = 0.025; and RMSEA = 0.068, CI 95% [0.034; 0.099]. The chi-squared difference test was significant (ΔSBχ^2^(1) = 59.3, *p* < 0.001), supporting the two-factor structure of the Functionality Satisfaction scale (see Supplementary Materials, Table S4). All items loaded strongly, ranging from 0.66 to 0.93 (see Figure 1). High McDonald’s omega values were found (Global score: ω = 0.94; Factor 1: ω = 0.94; Factor 2: ω = 0.87).

#### 3.2.2. Functionality Intent

**Figure 2:**
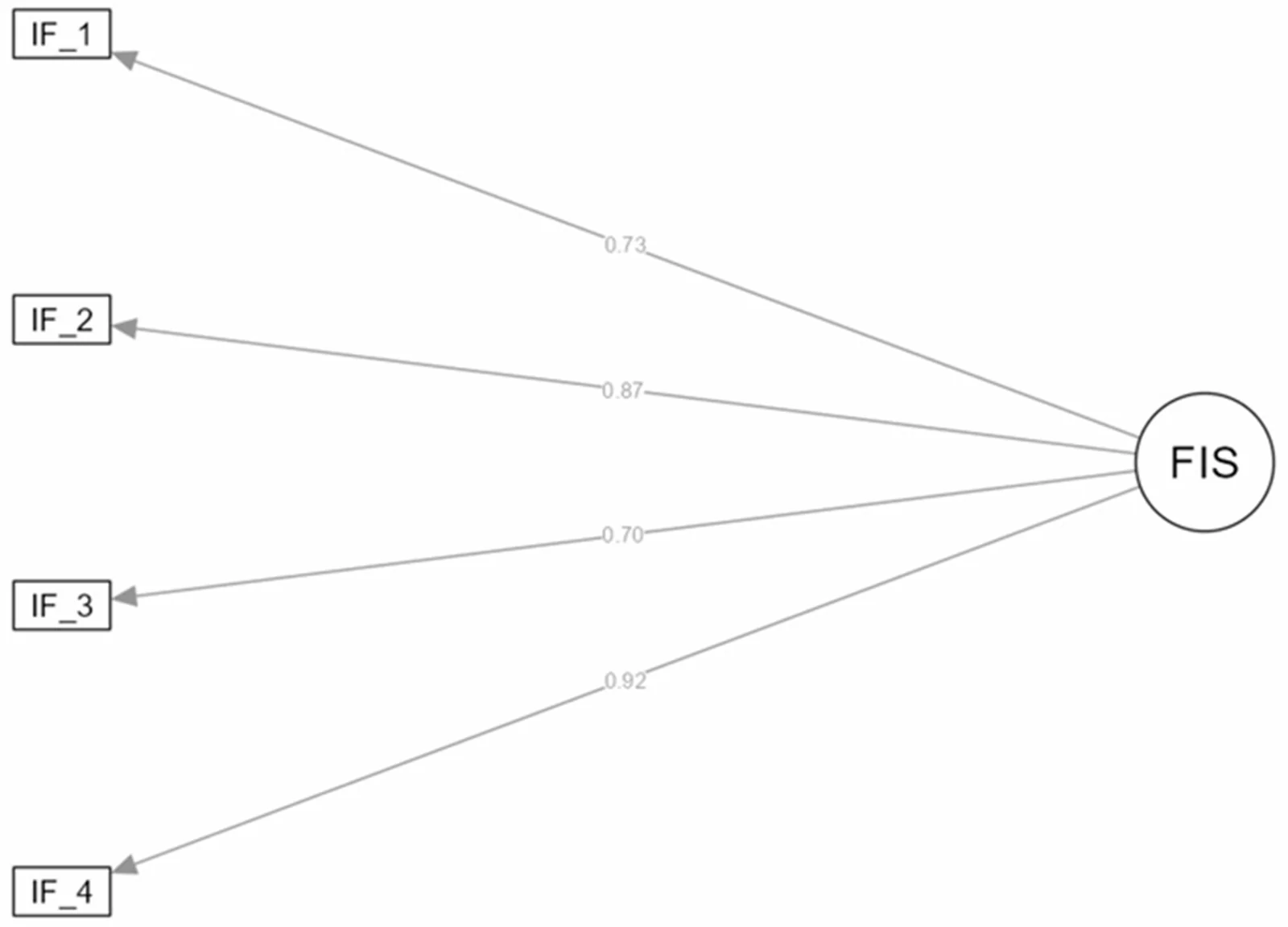
Path diagram and estimates for the unidimensional structure of Functionality Intent Scale scores in sample 2.

Confirmatory factor analysis. The two-factor model suggested an overall satisfactory fit: (i) SBχ^2^(1) = 2.81, *p* = 0.094, (ii) SBχ^2^/df = 2.81, (iii) CFI = 0.995, (iv) TLI = 0.967, and (v) SRMR = 0.019; the only exception was RMSEA = 0.110, CI 95% [0.000; 0.272]. The difference between the models was non-significant (ΔSBχ^2^(1) = 0.006, *p* = 0.94; see Supplementary Materials, Table S5). However, all the fit indices supported the unidimensional structure as a better fit (SBχ^2^(2) = 2.73, *p* = 0.256; SBχ^2^/df = 1.365; CFI = 0.998; TLI = 0.993; SRMR = 0.018; and RMSEA = 0.050, CI 95% [0.000; 0.180]): thus, the one-factor model solution was retained. This choice was also supported by the strong correlation between the two factors (β = 1.00), suggesting that they measure the same dimension. These modifications made during confirmatory analysis transformed it into an exploratory procedure. All items loaded strongly, ranging from 0.70 to 0.92 (see Figure 2). McDonald’s omega value was 0.88.

#### 3.2.3. Tests of Measurement Invariance Across Gender

To examine whether the FSS and FIS functioned equivalently for women and men, measurement invariance was tested using multi-group confirmatory factor analysis. Persons of diverse genders were removed from these analyses, due to insufficient sample size, resulting in the exclusion of nine participants.

##### Functionality Satisfaction Scale

The configural invariance model provided a good fit to the data (see Table 3), indicating that the factor structure was similar across women and men. The metric invariance model also showed satisfactory fit, and the comparison with the configural model was non-significant, ΔSBχ^2^(6) = 6.99, *p* = 0.322, with negligible changes in fit indices (ΔCFI = −0.001, ΔSRMR = 0.012, ΔRMSEA = −0.002). These findings support the equivalence of the item loadings on the factors (Putnick & Bornstein, 2016). The scalar invariance model likewise demonstrated acceptable fit. The comparison with the metric model yielded ΔSBχ^2^(6) = 11.85, *p* = 0.065, ΔCFI = −0.003, ΔSRMR = 0.003, ΔRMSEA = −0.002, supporting scalar invariance across gender. In contrast, the strict invariance model showed a significant worsening in fit relative to the scalar model, ΔSBχ^2^(8) = 49.63, *p* < 0.001, and the change in CFI was higher than the conventional cutoff (ΔCFI = −0.011), invalidating strict invariance.

##### Functionality Intent Scale

The configural model demonstrated excellent fit across women and men (see Table 4). Metric invariance was supported, as the comparison with the configural model was non-significant, ΔSBχ^2^(3) = 1.97, *p* = 0.579. Scalar invariance was also supported: the comparison with the metric model was non-significant, ΔSBχ^2^(3) = 0.51, *p* = 0.917, with no meaningful deterioration in fit. In contrast, strict invariance was not supported, as the strict model fit significantly worse than the scalar model, ΔSBχ^2^(4) = 110.79, *p* < 0.001, accompanied by a notable decline in CFI, ΔCFI = −0.030. In summary, the FIS demonstrated configural, metric, and scalar invariance across gender, but not strict invariance. These findings should nevertheless be interpreted cautiously given the preliminary status of the FIS factor structure.

#### 3.2.4. Tests of Measurement Invariance Across Age

Given the relatively young sample, we conducted an exploratory median split (Me = 25) to examine measurement invariance across age groups. Participants were therefore separated into two groups: emerging adults (18–25 years old, *n* = 141) and adults older than 25 years (*n* = 131).

##### Functionality Satisfaction Scale

The configural model showed good fit across age groups, indicating equivalent factor structure for emerging adults and adults (see Table 5). The model fit indices remained satisfactory after constraining the factor loadings (step 2) and after constraining item intercepts (step 3), and the comparison with the nested model was non-significant and showed minimal changes in fit indices. The strict invariance model, however, resulted in a significant decrement in fit, ΔSBχ^2^(8) = 18.89, *p* = 0.015, although the decline in CFI was modest (ΔCFI = −0.010) and the alternative fit indices remained satisfactory, suggesting weak support for strict invariance.

##### Functionality Intent Scale

For the FIS across age, the configural and metric models showed acceptable fit, and the comparison between them was non-significant, ΔSBχ^2^(3) = 1.15, *p* = 0.765, supporting configural and metric invariance across age (see Table 6). In contrast, scalar invariance was not supported. The scalar model showed a significant worsening in fit compared with the metric model, ΔSBχ^2^(3) = 17.18, *p* = 0.001, with meaningful changes in fit indices exceeding the criteria fixed by Chen (2007). Inspection of the individual equality constraints indicated that the lack of scalar invariance was primarily attributable to differences in the intercept of item 3 (χ^2^(1) = 10.82, *p* = 0.001) and, to a lesser extent, of item 1 (χ^2^(1) = 4.65, *p* = 0.031). These results suggest that items 1 and 3 functioned differently across emerging adults and adults, despite equivalent item loadings (metric invariance). The remaining intercept constraints were non-significant, indicating that items 2 and 4 functioned equivalently across age groups. The strict invariance model also showed poorer fit than the scalar model, ΔSBχ^2^(4) = 15.03, *p* = 0.005, with further deterioration in fit indices. Overall, the FIS demonstrated configural and metric invariance across age, but scalar and strict invariance were not supported. Consequently, comparisons of latent or observed mean scores should be interpreted with caution, as item intercepts and residual variances differ between emerging adults and adults older than 25 years.

### 3.3. Study 2 Discussion

The aim of this second study was to confirm the factor structures identified in study 1 for the scales of interest by using CFA in a different sample of French adults.

The findings revealed an eight-item, two-factor structure, with adequate fit for the FSS. We compared this model to the original unifactorial structure (Mulgrew & Courtney, 2022). The significance of the chi-squared difference test between the one-factor and two-factor models confirmed the two dimensions of this scale in our French adult sample. This is not surprising, as the theoretical aim of the FSS is to measure multiple facets of body functionality. However, it would be valuable to further explore the original scale structure in other samples, perhaps in subgroups (e.g., by gender), to better identify the origin of this divergence and confirm the two-factor structure. In the absence of further analyses, it is difficult to determine whether the structural difference stems from cultural variations in body functionality or whether it may be attributed to methodological discrepancies, as the original work employed only EFA (Mulgrew & Courtney, 2022). Additional testing of the Australian version may also help to understand whether the difference observed here between the French and the Australian version could be due to cultural differences in the conceptualization of body functionality.

The FIS revealed a satisfactory overall model fit and no significant difference from a one-factor structure. Nevertheless, a unidimensional factor solution seemed most relevant based on indicators of goodness of fit and the strong correlation between the two factors. While further analyses are needed to confirm whether this model is the superior solution (Swami & Barron, 2019), these findings led to the one-factor model being retained. Further examination of the factor structure in a separate sample is warranted.

Both scales demonstrated invariance across gender up to the scalar level, but not at the strict level. Across age groups, the FSS showed configural, metric, and scalar invariance, whereas the FIS was invariant only up to the metric level. This latter finding suggests that some observed age differences may reflect item intercept bias rather than only latent differences in the construct, which limits the validity of direct mean comparisons across age groups (Cheung & Lau, 2012; Putnick & Bornstein, 2016). Specifically, inspection of individual equality constraints indicated that the main source of non-invariance was located in the intercept of item 3 and, to a lesser extent, item 1. Emerging adults and adults may endorse these items differently even despite having similar levels of the latent construct (Putnick & Bornstein, 2016). Because item 3 emphasizes the cognitive reorientation from objectified appearance monitoring toward valuing what the body can do, one plausible explanation is that age groups differ in the relative importance they attribute to appearance versus functionality. This interpretation is aligned with developmental research showing that adolescence and emerging adulthood are periods in which appearance-related concerns become particularly central (Gendelman et al., 2026; Vangeel et al., 2018). Consequently, the shift from appearance focus to a functionality-oriented perspective may be interpreted differently by emerging adults than by adults older than 25 years. This pattern is also consistent with some recent findings of higher functionality appreciation levels among older adults than younger individuals (He et al., 2023; Maïano et al., 2026; Mebarak et al., 2024), although evidence on age differences remains mixed across the literature (Alleva & Tylka, 2021; Linardon et al., 2023). This age-related difference may reflect reduced endorsement of sociocultural pressures and greater life experience that facilitate placing greater value on body functionality over appearance (Maïano et al., 2026).

Item 1, which is behaviorally framed around pleasurable and stimulating movement, showed similar intercept non-invariance. One possible explanation is that wording centred on the physical and pleasurable aspects of body functionality may make responses more dependent on age-related lifestyle (activity patterns and opportunities) or bodily experience (current health, energy, physical limitations, for instance; He et al., 2023).

## 4. General Discussion

The aim of this study was to translate and examine the psychometric properties of the FSS and the FIS (Mulgrew & Courtney, 2022) among a French-speaking general population. Across two studies, exploratory and confirmatory analyses were conducted to evaluate the measures’ factor structures, along with assessments of reliability and convergent and divergent validity. Findings provided evidence supporting the reliability and validity of the FSS, while preliminary evidence was obtained for the FIS. Taken together, these results raise several points of interest regarding the clinical and scientific challenges in the conceptualization and assessment of body functionality. Furthermore, given the rising interest in body functionality as an index of positive body image and adaptive behaviours (Linardon et al., 2023), these measures will be useful to better characterize this construct and its cognitive and behavioural implications among French-speaking groups. Moreover, various interventions targeting body functionality have been developed to help promote positive body image, and measures of functionality are central to efforts to evaluate these interventions in French-speaking groups, including the Functionality Intent Scale (FIS; Mulgrew & Courtney, 2022).

Findings provided strong support for the psychometric structure of the FSS French translation, although the French version revealed a slightly different factor structure from the original version. Despite promising results, the FIS requires further independent cross-validation to confirm its unidimensional structure through CFA. In addition, findings supported the reliability and validity of both translated scales, notably through convergent validity with measures of positive body image and test–retest reliability.

Consistent with the findings on functionality appreciation, mixed results were found for the associations between the global scores of FSS and FIS on the one hand and BMI and age on the other hand. Similar results were found in numerous studies assessing the FAS (Alleva et al., 2017; Arnaud et al., 2025; Cerea et al., 2021; He et al., 2023; Maïano et al., 2026; Mebarak et al., 2024; Riechers & Warschburger, 2025; Swami et al., 2019, 2021b; Zamora et al., 2024). Together, these results suggest that the relationships between body functionality and demographic or weight-related variables remain complex and warrant further investigation. They also indicate that functionality-related experiences may not be entirely weight-neutral in practice, possibly in part because of internalized weight-stigma. Future research should examine the roles of BMI and internalized weight stigma in shaping functionality appreciation, especially through longitudinal studies.

Taken together, these findings yield important research and practice implications. First, the scales lay the groundwork for research aiming to replicate and extend work conducted in other cultural and geographic groups related to body functionality (Alleva et al., 2023; Urvelyte & Perminas, 2022). In particular, examinations of the cross-sectional and longitudinal associations between body functionality and dimensions of mental and physical health would be important. In addition, these scales allow for the examination of group invariances and the investigation of the ways in which body functionality may vary according to gender, sexual orientation, age, ability, or other relevant characteristics. In addition, as described above, body functionality has been identified as a promising target for interventions aiming to increase positive body image, and the availability of French-language versions of these scales will allow for their adaptation and further refinement.

In terms of practice implications, the robust associations between higher levels of functionality satisfaction and intent and other indices of positive body image, to a large extent independent of age and BMI, provide additional support for focusing on functionality with individuals at risk of body image concerns across a range of demographic groups. In addition, these scales may prove useful tools for increasing awareness of body functionality among a range of groups with mental and physical concerns or transitions.

## 5. Limitations

This study presents several important limitations. First, further work using qualitative and expert-based methodologies is warranted to more comprehensively establish content validity (Swami et al., 2021a). Second, while our translation procedure was designed to enhance semantic and conceptual equivalence, it did not include independent backtranslation, formal expert-content evaluation, or cognitive pilot testing with members of the target population, which should be considered when interpreting the cross-cultural adaptation process (Ambuehl & Inauen, 2022; Swami & Barron, 2019). The participants were predominantly women and relatively young, which restricts the generalizability of our findings. Future studies should validate the psychometric properties of these scales in more diverse samples, including more men and participants of diverse genders, a broader age range, and clinical populations. The absence of data on education level, disability status, chronic disease, or eating disorder history represents an important limitation, as these factors may affect body functionality. Additionally, no formal data quality screening procedures (attention checks, duplicate-response detection, or completion-time screening) were implemented. Future studies should incorporate systematic data quality procedures to strengthen the robustness of results. In addition, the results of CFA for the FIS should be interpreted carefully, as modifications were made from the initial model identified during EFA, changing the confirmatory nature of our analysis into an exploratory one (Swami & Barron, 2019), although multiple models were compared, which adds strength to our findings. Additional examinations of the psychometric properties of this scale should seek to replicate the current findings. Other measures, such as negative body image or eating disorder symptom measures, should be used to better assess divergent validity. Tests of predictive validity would also strengthen the validity evidence for these scales. We tested measurement invariance across gender, as establishing invariance is a necessary step for meaningful comparisons between groups (Swami & Barron, 2019). However, the relatively small and unequal sample size for men may have reduced the power of the invariance tests and should therefore be taken into account when interpreting the results. Moreover, invariance was examined only between women and men because the number of participants of diverse genders in our sample was too small for robust multi-group invariance testing. Future studies with larger and more balanced subgroup sizes, including multiple gender identities, would enable a more inclusive and psychometrically defensible assessment of whether the measure operates equivalently across these groups. It is worth noting that these studies involved dozens of statistical tests (correlations, hierarchical regressions, reliability analyses, *t*-tests, EFA, CFA, measurement invariance, model comparisons, etc.), which raises type I error risk (Nicholson et al., 2022).

## 6. Conclusions

This study provides evidence for the strong psychometric properties of the FSS in a sample of the French general population, while the FIS still requires independent cross-validation to confirm its unidimensional structure. The availability of the FSS and FIS in French fills an important gap in useful French-language scales for the assessment of body functionality. Moving forward, it would be interesting to explore whether functionality intent is associated with the adoption of adaptive and health-promoting behaviours that foster functionality awareness (such as intuitive eating, exposure to nature, involvement in creative and physical activities, for instance). Future research should incorporate behavioral, observational, or ecological momentary assessment indices to examine whether functionality intent predicts real-world behaviors. Additional work replicating these findings is warranted. Nevertheless, these scales pave the way for a program of research focused on body functionality among French-speaking groups.

## Figures and Tables

**Table 1:** Exploratory factor analysis of the Functionality Satisfaction Scale.

	EFA	
	Factor 1	Factor 2
1. Right now, I feel satisfied with what my body can do/Actuellement, je suis satisfait.e de ce que mon corps est capable de faire.	**0.79**	*0.11*
2. At present, I feel satisfied how my body can move/Actuellement, je suis satisfait.e de la façon dont mon corps peut bouger.	**0.95**	*−0.09*
3. Currently, I feel that my body is capable of many things/Actuellement, je sens que mon corps est capable de beaucoup de choses.	**0.85**	*0.07*
4. Right now, I feel respect for the many unique and varied tasks my body performs/Actuellement, j’éprouve du respect pour les nombreuses tâches uniques et variées que mon corps accomplit.	*0.30*	**0.52**
5. At this current moment, I feel that the physical capabilities of my body allow me to connect with the world in a meaningful way/En ce moment même, j’ai le sentiment que les capacités physiques de mon corps me permettent d’être en contact avec le monde de façon cohérente.	*0.11*	**0.78**
6. At present, I feel that my body helps me to communicate with others/Actuellement, j’ai le sentiment que mon corps m’aide à communiquer avec les autres.	*−0.11*	**0.86**
7. Right now, I feel that my body enables me to explore a diverse range of activities which I enjoy/Actuellement, j’ai le sentiment que mon corps me permet d’explorer une grande variété d’activités que j’aime.	**0.47**	0.42
8. Currently, I respect that my body has the ability to help me to rest, sleep, and restore/Actuellement, je respecte la capacité de mon corps à m’aider à me reposer, à dormir et à me rétablir.	*0.12*	**0.48**
% of the common variance	36.8	29.9
McDonald’s ω	0.92	0.83

Note: N = 528. Extraction method: Principal-axis factoring; Rotation: Oblimin. Coefficients lower than 0.40 are italicized and are not retained for the factor. Coefficients in bold are retained for the factor.

**Table 2:** Exploratory factor analysis of the Functionality Intent Scale.

	EFA	
	Factor 1	Factor 2
1. I intend to move my body in ways that I find physically stimulating and enjoyable/J’ai l’intention de bouger mon corps d’une manière que je trouve physiquement stimulante et agréable.	*−0.05*	**0.76**
2. I intend to be attentive to what my body needs/J’ai l’intention d’être attentif/attentive aux besoins de mon corps.	**0.55**	*0.31*
3. I intend to focus on appreciating the many functions of my body, rather than my appearance/J’ai l’intention de me concentrer sur la compréhension des nombreuses fonctions de mon corps plutôt que de me concentrer sur mon apparence.	**0.80**	*−0.06*
4. I intend to engage in self-care and other meaningful activities that help me to look after my body and mind/J’ai l’intention de prendre soin de moi et de m’engager dans des activités qui me semblent importantes et qui m’aident à prendre soin de mon corps et de mon esprit.	*0.38*	**0.53**
% of the common variance	31.9	28.4
McDonald’s ω	0.71	0.72

Note: N = 528. Extraction method: Principal-axis factoring; Rotation: Oblimin. Coefficients lower than 0.40 are italicized and are not retained for the factor. Coefficients in bold are retained for the factor.

**Table 3:** Assessment of measurement invariance for the FSS by gender.

Model	SBχ^2^ (df)	Robust CFI	Robust SRMR	Robust RMSEA	Model Comparison	ΔSBχ^2^ (Δdf)	*p*	Δrobust CFI	Δrobust SRMR	Δrobust RMSEA
Configural	55.32 (38)	0.988	0.029	0.059						
Metric	62.54 (44)	0.987	0.041	0.057	Configural vs. metric	6.99 (6)	0.322	−0.001	0.012	−0.002
Scalar	73.05 (50)	0.984	0.044	0.059	Metric vs. scalar	11.85 (6)	0.065	−0.003	0.003	0.002
Strict	97.83 (58)	0.973	0.041	0.072	Scalar vs. strict	49.63 (8)	<0.001	−0.011	−0.003	0.013

Note: SB = Satorra–Bentler; CFI = Comparative Fit Index; SRMR = Standardized Root Mean Square Residual; RMSEA = Root Mean Square Error of Approximation.

**Table 4:** Assessment of measurement invariance for the FIS by gender.

Model	SBχ^2^ (df)	Robust CFI	Robust SRMR	Robust RMSEA	Model Comparison	ΔSBχ^2^ (Δdf)	*p*	Δrobust CFI	Δrobust SRMR	Δrobust RMSEA
Configural	4.58 (4)	0.999	0.020	0.033						
Metric	7.10 (7)	1.000	0.032	0.010	Configural vs. metric	1.97 (3)	0.579	0.001	0.012	−0.023
Scalar	8.42 (10)	1.000	0.033	0.000	Metric vs. scalar	0.51 (3)	0.917	0.000	0.001	−0.010
Strict	25.84 (14)	0.970	0.036	0.080	Scalar vs. strict	110.79 (4)	<0.001	−0.030	0.003	0.080

Note: SB = Satorra–Bentler; CFI = Comparative Fit Index; SRMR = Standardized Root Mean Square Residual; RMSEA = Root Mean Square Error of Approximation.

**Table 5:** Assessment of measurement invariance for the FSS by age.

Model	SBχ^2^ (df)	Robust CFI	Robust SRMR	Robust RMSEA	Model Comparison	ΔSBχ^2^ (Δdf)	*p*	Δrobust CFI	Δrobust SRMR	Δrobust RMSEA
Configural	51.25 (38)	0.991	0.029	0.058						
Metric	58.77 (44)	0.990	0.044	0.055	Configural vs. metric	7.32 (6)	0.293	−0.001	0.015	−0.002
Scalar	67.37 (50)	0.989	0.046	0.055	Metric vs. scalar	8.79 (6)	0.186	−0.001	0.002	0.000
Strict	89.03 (58)	0.979	0.049	0.071	Scalar vs. strict	18.89 (8)	0.015	−0.010	0.003	0.015

Note: SB = Satorra–Bentler; CFI = Comparative Fit Index; SRMR = Standardized Root Mean Square Residual; RMSEA = Root Mean Square Error of Approximation.

**Table 6:** Assessment of measurement invariance for the FIS by age.

Model	SBχ^2^ (df)	Robust CFI	Robust SRMR	Robust RMSEA	Model Comparison	ΔSBχ^2^ (Δdf)	*p*	Δrobust CFI	Δrobust SRMR	Δrobust RMSEA
Configural	6.84 (4)	0.992	0.019	0.094						
Metric	9.04 (7)	0.995	0.028	0.055	Configural vs. metric	1.15 (3)	0.765	0.003	0.009	−0.039
Scalar	22.16 (10)	0.976	0.054	0.106	Metric vs. scalar	17.19 (3)	0.001	−0.019	0.026	0.051
Strict	38.49 (14)	0.947	0.063	0.133	Scalar vs. strict	15.03 (4)	0.005	−0.029	0.009	0.027

Note: SB = Satorra–Bentler; CFI = Comparative Fit Index; SRMR = Standardized Root Mean Square Residual; RMSEA = Root Mean Square Error of Approximation.

## Data Availability

The datasets used for this research are available on request from the corresponding author.

## Supplementary Materials

The following supporting information can be downloaded at: https://www.mdpi.com/article/10.3390/ejihpe16090137/s1. Appendix S1: Final French version of the Functionality Satisfaction Scale. Appendix S2: Final French version of the Functionality Intent Scale. Supplementary Table S1: Functionality Satisfaction Scale inter-item correlations and item-total correlations (ITC). Supplementary Table S2: Correlations between the scores of the different scales, BMI, and age. Supplementary Table S3: Functionality Satisfaction Scale inter-item correlations and item-total correlations (ITC). Supplementary Table S4: Comparison of the hypothesized and alternative models for the Functionality Satisfaction Scale. Supplementary Table S5: Comparison of the hypothesized and alternative models for the Functionality Intent Scale.
