# Supplementary material for: Translation and Validation of the Functionality Satisfaction and the Functionality Intent Scales in a French Sample
Source: Eur J Investig Health Psychol Educ. 2026 Sep 9;16(9):137. doi: 10.3390/ejihpe16090137 (PMC13605285; doi:10.3390/ejihpe16090137)
Supplement: Supplementary file 1 [file ejihpe-16-00137-s001.zip › ejihpe-4415547-supplementary.pdf]

Appendix S1: Final French version of the Functionality Satisfaction Scale

Consigne :

Considérez comment vous vous sentez actuellement et indiquer la réponse de votre choix à l'aide du curseur, allant de 0 (pas du tout) à 100 (totalement).

1. *Actuellement, je suis satisfait.e de ce que mon corps est capable de faire*

2. *Actuellement, je suis satisfait.e de la façon dont mon corps peut bouger*

3. *Actuellement, je sens que mon corps est capable de beaucoup de choses*

4. *Actuellement, j'éprouve du respect pour les nombreuses tâches uniques et variées que mon corps accomplit*

5. *En ce moment même, j'ai le sentiment que les capacités physiques de mon corps me permettent d'être en contact avec le monde de façon cohérente*

6. *Actuellement, j'ai le sentiment que mon corps m'aide à communiquer avec les autres*

7. *Actuellement, j'ai le sentiment que mon corps me permet d'explorer une grande variété d'activités que j'aime*

8. *Actuellement, je respecte la capacité de mon corps à m'aider à me reposer, à dormir et à me rétablir*

Scoring procedure:

Subscale 1 "Satisfaction with body capacities": mean of items 1, 2, 3, and 7

Subscale 2 "Gratitude for body functions and worldly connection": mean of items 4, 5, 6, and 8

Global score: mean of the responses

Appendix S2: Final French version of the Functionality Intent Scale

Consigne :

Considérez comment vous vous sentez actuellement et indiquer la réponse de votre choix à l'aide du curseur, allant de 0 (pas du tout) à 100 (totalement).

1. *J'ai l'intention de bouger mon corps d'une manière que je trouve physiquement stimulante et agréable.*

2. *J'ai l'intention d'être attentif/attentive aux besoins de mon corps.*

3. *J'ai l'intention de me concentrer sur la compréhension des nombreuses fonctions de mon corps plutôt que de me concentrer sur mon apparence.*

4. *J'ai l'intention de prendre soin de moi et de m'engager dans des activités qui me semblent importantes et qui m'aident à prendre soin de mon corps et de mon esprit.*

Scoring procedure:

Global score: mean of the responses

Supplementary materials:

Table S1

Functionality Satisfaction Scale inter-item correlations and item-total correlations (ITC)

	Item 1	Item 2	Item 3	Item 4	Item 5	Item 6	Item 7	Item 8	ITC
Item 1	-								
Item 2	$.78^{***}$	-							
Item 3	$.77^{***}$	$.80^{***}$	-						
Item 4	$.63^{***}$	$.57^{***}$	$.65^{***}$	-					
Item 5	$.65^{***}$	$.61^{***}$	$.66^{***}$	$.64^{***}$	-				
Item 6	$.52^{***}$	$.48^{***}$	$.52^{***}$	$.54^{***}$	$.70^{***}$	-			
Item 7	$.70^{***}$	$.67^{***}$	$.74^{***}$	$.64^{***}$	$.73^{***}$	$.55^{***}$	-		
Item 8	$.45^{***}$	$.39^{***}$	$.45^{***}$	$.51^{***}$	$.45^{***}$	$.44^{***}$	$.48^{***}$		
ITC	$.84^{***}$	$.81^{***}$	$.86^{***}$	$.80^{***}$	$.85^{***}$	$.75^{***}$	$.86^{***}$	$.66^{***}$	-

Note: *** $p < .001$

Table S2

Correlations between the scores of the different scales, BMI, and age

	(1)	(2)	(3)	(4)	(5)	(6)	(7)
(1) Functionality Satisfaction	-						
(2) Functionality Intent	.56***						
(3) Functionality Appreciation (FAS)	.72***	.52***	-				
(4) Body Appreciation (BAS-2)	.68***	.55***	.69***	-			
(5) Body esteem (BES)	.59***	.42***	.55***	.84***	-		
(6) BMI	-.23***	-.04	-.21***	-.24***	-.35***	-	
(7) Age	.09*	.12**	.05	.12**	.08	.24***	-

Note: * $p < .05$; ** $p < .01$; *** $p < .001$

Table S3

Functionality Satisfaction Scale inter-item correlations and item-total correlations (ITC)

	Item 1	Item 2	Item 3	Item 4	ITC
Item 1	-				
Item 2	.47***	-			
Item 3	.32***	.57***	-		
Item 4	.55***	.64***	.54***	-	
ITC	.73***	.83***	.77***	.86***	-

Note: *** p <.001

Table S4

Comparison of the hypothesized and alternative models for the Functionality Satisfaction Scale.

Model	SBχ^2	df	Sbχ^2/df	Robust CFI	Robust TLI	Robust SRMR	Robust RMSEA	ΔSBχ^2
Two-factor model	37.4^{**}	19	1.968	0.987	0.981	0.025	0.068	
One-factor model	96.7^{***}	20	4.835	0.948	0.927	0.039	0.133	136^{***}

Note: SB= Satorra–Bentler; CFI= Comparative Fit Index; TLI= Tucker–Lewis Index; SRMR= Standardized Root Mean Square Residual; RMSEA= Root Mean Square Error of Approximation; $^{*}p < .05$; $^{**} p < .01$; $^{***}p < .001$

Table S5

Comparison of the hypothesized and alternative models for the Functionality Intent Scale.

Model	SBχ^2	df	Sbχ^2/ df	Robust CFI	Robust TLI	Robust SRMR	Robust RMSEA	ΔSBχ^2
Two-factor model	2.81	1	2.81	0.995	0.967	0.019	0.110	
One-factor model	2.73	2	1.365	0.998	0.993	0.018	0.050	0.006

Note: SB= Satorra–Bentler; CFI= Comparative Fit Index; TLI= Tucker–Lewis Index; SRMR= Standardized Root Mean Square Residual; RMSEA= Root Mean Square Error of Approximation; $^{*}p < .05$; $^{**} p < .01$; $^{***}p < .001$
